# Influence of pH and Heat Treatment on the Physicochemical, Interfacial, and Emulsifying Properties of Hemp Seed Protein Dispersions

**DOI:** 10.3390/foods15020257

**Published:** 2026-01-10

**Authors:** Davide Odelli, Lingxin You, Jennyfer Fortuin, Jérôme Bour, Marcus Iken, Axel Archaimbault, Christos Soukoulis

**Affiliations:** 1 Environmental Research and Innovation (ERIN) Department, Luxembourg Institute of Science and Technology (LIST), 5 Avenue des Hauts Fourneaux, L-4362 Esch-sur-Alzette, Luxembourg; 2 Institute of Food, Nutrition and Health, ETH Zurich, 8092 Zurich, Switzerland; 3 Food Quality and Design Group, Wageningen University and Research, 6708 NL Wageningen, The Netherlands; 4 PM-International AG, 15 Wäistrooss, L-5445 Schengen, Luxembourg

**Keywords:** hemp seed protein isolate dispersions, interfacial adsorption, interfacial rheology, emulsifying properties, protein secondary structure

## Abstract

This study reports the effect of pH (2, 7, 10) and heat treatment (80 °C for 30 min) on the oil–water (o/w) interfacial behavior of hemp seed protein isolate (HPI) aqueous dispersions. The physicochemical, interfacial adsorption, rheology, and emulsifying properties of protein dispersions were evaluated. HPI dispersions at pH 10 exhibited the highest water solubility (60%), the greatest net charge (−27 mV), and the lowest hydrophobicity (~5 a.u.), promoting o/w interfacial pressure (π) and interfacial viscoelasticity. Strong interfacial viscoelastic protein layers (E^*^ = 25 mN/m) were also observed under acidic conditions (pH 2), where proteins exhibited high solubility (40%), a high positive net charge (21 mV), and increased hydrophobicity (46 a.u.). HPI dispersions in their neutral state (pH 7) were not able to form stable o/w emulsions due to their poor physicochemical properties such as low solubility (18%), low surface charge (−18 mV), and hydrophobicity (~5 a.u.). Heat treatment significantly increased the charge and hydrophobicity of both neutral and alkaline proteins (~30 mV and ~10 a.u., respectively), increasing their particle size distribution and ultimately reducing their interfacial protein layer elasticity (E^*^ = 20 and 13 nM/m, respectively). While particles at acidic conditions showed high thermal resistance, heat treatment improved the emulsifying stability in alkaline conditions while further reducing it in the neutral state. Overall, HPI dispersions demonstrated the ability to form stable emulsions at both alkaline and acid pHs, with those formed at pH 2 exhibiting a lower droplet size and superior stability.

## 1. Introduction

In response to the growing consumer preference for natural, wholesome, and clean-label products, the food industry has increasingly adopted ingredients (e.g., stabilizers, emulsifiers, proteins, soluble fibers, etc.) that are perceived as natural, healthy, and sustainable. Emulsifiers are widely employed in food formulations as surface-active materials to improve the stability and extend the shelf-life of colloidal food systems such as foams, emulsions, and bigels [1]. Among the various classes of food-grade emulsifiers, proteins and their hydrolysates present distinct advantages over synthetic emulsifiers, including superior emulsion stability, irreversible adsorption at the oil–water interface, and the ability to form thick, viscoelastic interfacial layers that enhance the thermodynamic and kinetic stability of food colloids. Furthermore, their natural origin, biocompatibility, biodegradability, cost-effectiveness, and wide availability align well with the growing demand for sustainable and clean-label food products [2,3,4].

It is well-documented that extrinsic physicochemical parameters such as pH, ionic strength, and temperature have a considerable influence on proteins’ structures and intermolecular interactions, ultimately affecting the preparation and storage of emulsion-based food products [5]. Moreover, proteins’ adsorption kinetics onto the oil–water interface, as well as the mechanical elasticity of the interfacial layer, are directly related to the stability of emulsion droplets against destabilizing effects such as creaming, coalescence, flocculation, and Ostwald ripening [6]. Thus, a comprehensive understanding of the influence of environmental and processing conditions on the physicochemical and interfacial properties of proteins is fundamental for their employment as efficient emulsifiers in food products.

Hemp (*Cannabis sativa* L.) seeds are an emerging sustainable, alternative food bioresource rich in proteins, soluble and insoluble dietary fibers, omega-3 lipids, vitamins (vitamins E, D, and A), polyphenols, and terpenoids. They possess a multifaceted health benefit-conferring profile, including antioxidant, anti-inflammatory, hypocholesterolemic, and antimicrobial properties [7,8]. Hemp seed protein isolate consists of approximately 65% edestin (a globulin-type protein) and 33% albumin, is rich in all essential amino acids, and is particularly abundant in arginine and glutamine [9]. Previous studies have demonstrated that the extraction and isolation conditions are inextricably associated not only with the amino acid profile of hemp protein isolates, but also with their technofunctional, texturing, and interface-stabilizing properties [10,11]. Owing to its favorable nutritional profile and amphiphilic nature, hemp seed protein isolate (HPI) has also been assessed as a potential food-grade surface-active material for the stabilization of food emulsions [10,12,13]. A comparison among hemp seed protein fractions showed that the 2S albumin fraction exhibits better emulsifying capacity than the 7S and 11S fractions because its smaller polypeptide size allows for rapid and efficient rearrangement at the oil–water interface, while its higher sulfhydryl content promotes the formation of stronger disulfide-bonded interfacial membranes [14]. In addition, the pre-treatment of hemp seed protein isolate dispersions using high-intensity ultrasound, heat-aided alkaline pH-shifting, or antisolvent precipitation has been successfully employed to reduce the particle size, enhance solubility, surface-charge density, and hydrophobicity of hemp protein molecules, ultimately improving their adsorption and stabilizing properties at the oil–water interface [9,15,16].

Nonetheless, the effect of different pH conditions, as well as their combinations with heat treatment on hemp seed protein isolate (HPI)’s physicochemical, interfacial, and rheological properties at the oil–water interface, is still not complete. In order to employ HPI as a functional ingredient in actual food systems, a rigorous evaluation of its physicochemical and interfacial properties in all the possible food processing and formulation conditions (i.e., acidic, neutral, and alkaline environment, before and after heating) is needed.

Therefore, to provide a deeper understanding of HPI’s potential as a natural and clean-label emulsifier, in this study we investigated the effect of pH (2, 7, and 10) and heat treatment (80 °C for 30 min) on HPI-based dispersions to promote structural rearrangements, as well as surface-active properties. The obtained protein molecules were then assessed for their ability to adsorb onto the oil–water interface and create a viscoelastic membrane through interfacial rheology measurements. Specifically, we hypothesized that combinations of pH and heat treatment could significantly influence HPI dispersions’ physicochemical properties and, consequently, their kinetics of adsorption and viscoelasticity at the o/w interface, ultimately expanding their application as emulsifying agents.

## 2. Materials and Methods

### 2.1. Materials

Hemp seed meal (HSM) was kindly donated by All Organic Treasures GmbH (Am Muhlbach 38, Wiggensbach, Germany). Medium-chain triglyceride MCT coconut oil was purchased from Natures Aid Ltd. (Preston, UK). All of the chemicals used were of analytical grade.

### 2.2. Preparation of Hemp Seed Protein Isolate (HPI)

Hemp seed meal was serially oil-extracted using n-hexane (the HSM to n-hexane ratio was adjusted to 1:10 for each extraction step) to achieve a residual oil content at ~1% (*w*/*w*). Defatted HSM was then washed to remove phenolic compounds and other soluble components following the method described by Jia et al. [17]. Briefly, the HSM was mixed with a 40% (*v*/*v*) aqueous ethanol solution at a solid-to-liquid ratio of 1:5 (*w*/*w*). The dispersion was stirred at room temperature (20 ± 2 °C) for 20 min and then centrifuged at 6000 rpm for 10 min at 20 °C, after which the wet pellet was collected and the supernatant was discarded. This washing procedure was repeated at least twice, and the final pellet was dried in an oven at 40 °C overnight to ensure complete solvent evaporation.

Defatted and polyphenol-depleted HSM was dispersed in MilliQ water at a solid-to-liquid ratio of 1:10 (*w*/*w*). The HSM dispersion was adjusted to pH 10 using 1 M NaOH to promote protein dissolution under mechanical stirring for 2 h at ambient temperature. The resulting supernatant following centrifugation at 11,000 rpm for 15 min was precipitated at the isoelectric point of hemp seed proteins (≈5.1 ± 0.2), washed twice using MilliQ water, dialyzed (membrane cut-off M_w_ 12.3 kDa) against MilliQ water for at least 48 h, and subsequently freeze-dried (Alpha 1-2 LD Plus, Christ, Germany). The obtained powder was designated as hemp protein isolate (HPI). The nitrogen content of HPI was determined by the Dumas combustion method using an elemental analyzer (CHNS; Elementar Vario Cube, Langensenbold, Germany). The nitrogen content of the HPI was quantified by the Dumas combustion method by using an Elemental Analyzer (CHNS) (Elementar Vario Cube, Langensenbold, Germany). The HPI protein content was estimated at 91 ± 0.5 *w*/*w* after freeze-drying based on a nitrogen conversion factor of 5.7 [18].

### 2.3. HPI Dispersions Preparation

Protein dispersions were prepared by dissolving HPI powder (1.0% *w*/*w*) into Milli-Q water (18.2 mΩ, Millipore Inc., Burlington, MA, USA). The protein dispersions were mechanically stirred for 2 h at room temperature (20 ± 2 °C), and their pH was continuously adjusted to 2, 7, or 10 using either 1.0 M HCl or 1.0 M NaOH. The obtained samples are hereafter referred to as HPI2, HPI7, and HPI10 for samples at pH 2, 7, and 10, respectively. Heat treatment was subsequently applied to these samples, which were heated at 80 °C for 30 min followed by immediate cooling to 4 °C in an ice bath. The samples obtained after this process are hereafter referred to as HHPI2, HHPI7, and HHPI10 in order to refer to their pH and heat treatment conditions. All measurements were then conducted at ambient temperature.

### 2.4. Soluble Nitrogen Index

HPI soluble fraction was measured as a function of pH using the bicinchoninic acid (BCA) protein assay. Protein dispersions were centrifuged at 10,000× *g* for 10 min. Soluble nitrogen index was then obtained from the following equation, and the results are presented here as the mean of three different replicates:

(1) $$\mathrm{Soluble}\;\mathrm{nitrogen}\;\mathrm{index}\;\left(\%\right)\;=\;\frac{\mathrm{Protein}\;\mathrm{content}\;\mathrm{in}\;\mathrm{supernatant}}{\mathrm{Protein}\;\mathrm{content}\;\mathrm{in}\;\mathrm{initial}\;\mathrm{solution}}\;*\;100$$

### 2.5. Surface Charge Density

The zeta potential (ζ) of hemp seed protein dispersions was measured using a Zetasizer Nano Series (Malvern Instruments, Malvern, UK). The measurements were conducted at 25 °C applying a voltage of 50 V. The values were then calculated with the Henry equations, as follows:

(2) $$\zeta \;=\;\frac{3\eta \mu }{2\varepsilon f\left(\kappa R_{h}\right)}$$

where μ is the electrophoretic mobility (V Pa^−1^ s^−1^), η is the solvent viscosity (Pa s^−1^), ε is the medium dielectric constant (dimensionless), κ is the Debye length or the thickness of the double electric layer around the molecules (nm), and R_h_ is the hydrodynamic radius (nm). A value of 1.5 was used for f(κR_h_), which is the Henry’s constant, according to the Smoluchowski approximation. All the samples were measured in triplicate.

### 2.6. Surface Hydrophobicity

Protein surface hydrophobicity was measured as a function of pH before and after heating treatment using the method described by Kato et al. [19], with slight modifications, which is based on the fluorescence intensity given by the interaction between 8-anilino and 1-naphthalenesulfonate (ANS) and the hydrophobic region on the protein’s surface. In brief, a protein stock solution of 0.1% (*w*/*w*) was diluted to 0.025, 0.05, and 0.075% (*w*/*w*) with Milli-Q water. An amount of 180 µL of 50 µM ANS solution was mixed with 20 µL of protein solutions into 96-well microplates (LUMITRAC, Greiner Bio-one, Frickenhausen, Germany) and kept in the dark for 15 min. The fluorescence intensity (FI) of the samples was then measured using a Microplate reader (TECAN, Spark 20M_2, Männedorf, Switzerland) with an excitation wavelength between 360 and 390 nm and an emission wavelength between 470 and 500 nm. Net FI was calculated by subtracting blank FI values from the FI value of all the samples where proteins were mixed with ANS. An index of relative surface hydrophobicity (H_0_) was obtained as the slope of the net FI versus the protein concentration of all the samples. All measurements were reported as the mean of three independent replicates.

### 2.7. Protein Secondary Structure

Fourier transform infrared spectra (FTIR) were recorded at room temperature using an Optics Vertex spectrometer (Bruker, Billerica, MA, USA) in the Attenuated Total Reflectance (ATR) mode with a diamond crystal. Fifty scans were performed for each spectrum. All spectra were analyzed within the wavenumber range of 4000–500 cm^−1^. For the analysis of the secondary conformational stage of the protein, the amide I region (1600–1700 cm^−1^) was deconvoluted using Origin2019b software. Background-corrected spectra were obtained and analyzed by the second derivative of the amide I band regions for their component compositions and peak frequencies. The second derivative was performed using the Savitzky–Golay derivative routine, with parameter values of 8 data points with polynomial order 3, as this condition proved to be more informative. For the analysis, each HPI sample after preparation was freeze-dried, and approximately 200 mg of sample powder was employed. All the samples were measured in triplicate.

### 2.8. Dispersion Particle Size Distribution

Particle size distributions of the HPI samples were measured with a Mastersizer 3000 laser light-scattering equipment (Malvern Instruments, Worcestershire, UK) coupled with a Hydro MV sample handling unit. Milli-Q water was used as the dispersant, and freshly made dispersion samples were added until reaching an obscuration value between 4 and 10%. The particle size distribution was computed using the relative refractive index of (1.098), obtained from the ratio of the protein refractive index (1.46) and the refractive index of the dispersant (1.33). All measurements were reported as the mean of three independent replicates.

### 2.9. Interfacial Adsorption

Protein adsorption at the o/w interface can be condensed in three steps: (i) protein migration from the bulk to the interface, (ii) protein penetration and unfolding at the interface, and (iii) protein rearrangement to form an adsorbed layer into a more energetically favorable conformation [20]. Each of these steps can be used to describe the interfacial behavior of surface-active materials such as proteins. The kinetics of adsorption of the protein have been monitored, measuring the evolution of the interfacial pressure (π) according to Beverung et al. [21], where π is defined as the evolution of interfacial tension of a protein-stabilized interface in comparison with a pure o/w interface without any surface-active molecules, and it can be expressed as π = γ_0_ − γ, where γ_0_ is the interfacial tension of the pure o/w interface and γ is the interfacial tension of the adsorbed protein at the interface. Interfacial tension of protein samples was measured using an automated drop tensiometer (Tracker^TM^, Teclis Scientific, Civrieux-d’Azergues, France) equipped with an optical glass cuvette. The samples were diluted to a HPI concentration of 0.1 g/L and loaded into a syringe of 500 µL. A pendant drop with volume of 28 µL was formed on the tip of a pipetting needle (Cadence Science^TM^ 7939 G18, length of 10 cm, internal diameter of 1.2 mm, Staunton, VA, USA) in the MCT coconut oil phase. The interfacial tension was measured for 7200 s while keeping the droplet volume constant, allowing for protein adsorption at the oil–water interface until equilibrium was reached. Interfacial tension values were calculated from the droplet shape using the Laplace equation.

The first step of protein adsorption can be described by the Ward and Tordai model [22] as follows:

(3) $$\pi \;=\;2C_{0}\mathrm{kT}{\left(\frac{D_{t}}{3.14}\right)}^{1/2}$$

where C_0_ is the protein concentration in the dispersion, k is the Boltzmann constant and T is the absolute temperature, D is the diffusion coefficient, and t is the time. This model is based on the assumptions that the migration of the proteins to the interface is diffusion-controlled; there is no energy barrier to adsorption during migration; the protein conformation does not change after adsorption [6]. The initial plot of π vs. t^1/2^ gives a linear region which slope signifies the rate of initial diffusion-controlled migration (k_diff_) [23].

After proteins migrate to the interface, the rate of penetration, unfolding, and rearrangements of HPI can be obtained by the following first-order equation [24]:

(4) $$\ln\frac{\pi_{7200}\;-\;\pi_{t}}{\pi_{7200}\;-\;\pi_{0}}=\;{-k}_{i}t$$

where π_0_, π_t_, and π_7200_ are the interfacial pressure values at time 0, at any experimental time point, and at the final adsorption time point (7200 s), respectively. k_i_ is the first-order rate constant from which the plot of $\ln\frac{\pi_{7200}-{\;\pi }_{t}}{\pi_{7200\;}-\;\pi_{0}}$ as a function of time yields two linear regions. The first slope corresponds to the constant of penetration and unfolding k_u_, while the second slope to the constant of rearrangement (k_r_). All the samples were measured in triplicate.

### 2.10. Interfacial Rheology

The interfacial rheology of HPI samples was measured right after the interfacial tension measurement of the same droplet, assuring that the protein molecules reached the equilibrium phase at the interface. With the same equipment (Tracker^TM^, Teclis Scientific, Civrieux-d’Azergues, France), oscillatory dilatational rheological properties were evaluated by performing a continuous amplitude sweep test in which the droplet area was varied at a deformation amplitude of 10% and a constant frequency of 0.1 Hz, ensuring that all samples remained within the linear viscoelastic region (LVR). All measurements were conducted in triplicate at 25 °C.

### 2.11. Emulsion Preparation

Oil-in-water emulsions were prepared by homogenizing 1.0% (*w*/*w*) protein solutions with medium-chain triglyceride MCT coconut oil following the method described by You et al. [25], with slight modifications. In brief, 25 g of 1.0% (*w*/*w*) protein solutions and 1.25 mL of MCT coconut oil were homogenized using an ultrasonic processor (Sonics, Newtown, CT, USA) at 100 W for 1 min to prepare 5.0% (*w*/*v*) oil-in-water emulsions in a 50 mL falcon tube.

### 2.12. Droplet Size Distribution

The droplet size distribution of freshly made emulsion samples was measured with a Mastersizer 3000 laser light-scattering equipment (Malvern Instruments, Worcestershire, UK) coupled with a Hydro MV sample handling unit. Milli-Q water was used as the dispersant, and the emulsion samples were added until reaching an obscuration value between 4 and 10%. The droplet size was computed using the relative refractive index of (1.082), obtained from the ratio of the refractive index of MCT coconut oil (1.44) and the refractive index of the dispersant (1.33). The results were reported as the mean of three independent measurements.

### 2.13. Emulsion Stability

The rapid stability method was utilized by using a LUMiSizer Dispersion Analyser (L.U.M. GmbH, Berlin, Germany). The measurements were conducted applying centrifugal sedimentation in order to accelerate instability phenomena of each emulsion such as sedimentation, creaming, and/or flocculation. LUMiSizer parameters were stabilized at 25 °C under centrifugation at 4000 rpm for 10 min, as described by You et al. [25]. Oil droplet images of the emulsions were then monitored by CLSM. Measurements were performed in triplicate.

### 2.14. Statistical Analysis

The obtained results are expressed as the mean values of the replicated analysis. One-way analysis of variance (ANOVA) coupled with Tukey’s post hoc pairwise comparison test was conducted to assess significant differences among the mean values at a significance level of 0.05.

## 3. Results and Discussion

### 3.1. Physicochemical Properties of HPI Dispersions

#### 3.1.1. Soluble Nitrogen Index, Zeta Potential, and Hydrophobicity

**Figure 1:**
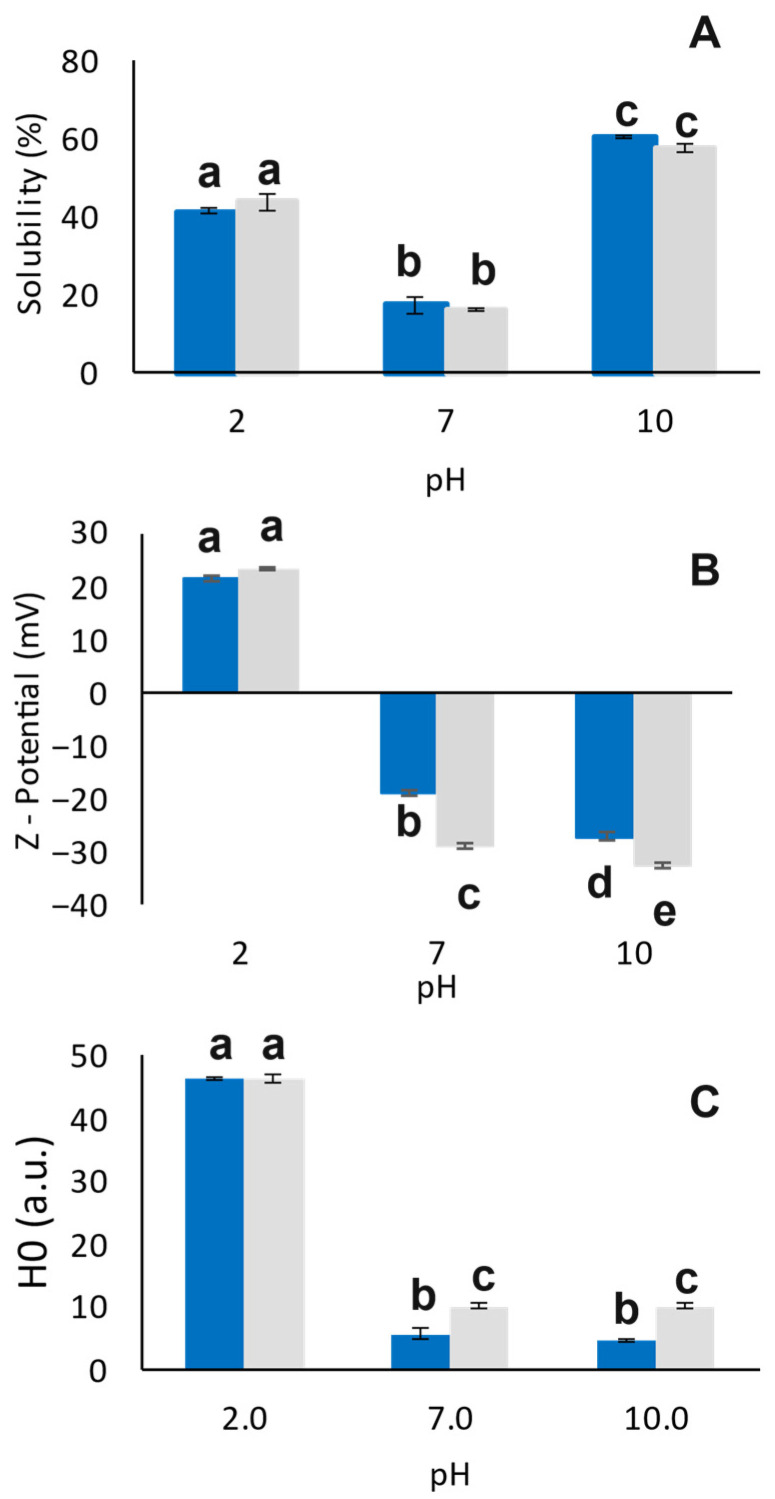
HPI soluble nitrogen index (%) (**A**), zeta potential (mV) (**B**), and surface hydrophobicity (arbitrary units, a.u.) (**C**) as a function of pH before and after heat treatment. Blue columns represent native protein particles, while gray columns heat-treated particles. ^a–e^ Different letters denote significant differences according to Tukey’s post hoc means comparison test (*p* < 0.05).

According to DLVO theory, the interfacial behavior of proteins is dictated by the interplay between surface charge, solubility, and hydrophobicity, which collectively modulate the balance between electrostatic repulsion and van der Waals attraction, thereby governing protein adsorption, structural rearrangement, and film stability at the oil–water interface [23]. Figure 1 illustrates the soluble nitrogen index (NSI), surface charge density, and hydrophobicity of HPI proteins and the obtained heat-induced proteins at different pH conditions. As expected, the soluble fraction of hemp seed protein isolate exhibited the characteristic “U-shaped” curve reported for several plant proteins, including soy, pea, chickpea, and faba bean (Figure 1A). HPI was mostly soluble at highly alkaline and acidic conditions (NSI was estimated at 60% and 41%, respectively), while the NSI markedly declined at neutral conditions (18%). These results are in keeping with the findings of Liu et al. [26], who reported a solubility plateau below 20% for HPI aqueous dispersions between pH 4 and 7, and with Shen et al. [27], who observed soluble fractions up to 80% at pH 9, confirming that HPI exerts a typical alkali-soluble protein behavior. Interestingly, heat treatment of the HPI dispersions did not significantly affect (*p* > 0.05) the percentage of the soluble protein fraction at any of the tested pH values, as the same solubility trend was maintained. Although heat treatment is commonly associated with protein denaturation and aggregation, such structural changes do not necessarily result in a measurable loss of soluble protein. Under the conditions investigated in this study, heating likely induced partial unfolding and aggregation of hemp proteins; however, insoluble aggregates were removed during the centrifugation step prior to solubility determination, while proteins remaining in the soluble or colloidal phase were still quantified by the BCA assay. Consequently, the concentration of proteins present in the soluble fraction was not significantly altered by heating. Similar behavior was reported by Liu et al. [28], who observed only minimal changes in the soluble fraction of hemp protein heated between 20 and 80 °C at pH 7. The authors attributed this response to the intrinsic characteristics of hemp protein, as well as to the specific extraction method and protein composition, which may confer resistance of the soluble fraction to heat-induced solubility losses.

As shown in Figure 1B, the ζ-potential of HPI dispersions shifted from positive values at acidic pH to negative values at neutral and alkaline pH, reflecting a different ionization of amino acid residues on protein surface. In particular, HPI10 exhibited the highest net surface charge (−27.1 mV), HPI7 showed the lowest (−18.8 mV), and HPI2 had an intermediate value (21.6 mV). Heating significantly increased (*p* < 0.05) the negative charge of both HHPI7 (−29.0 mV) and HHPI10 (−32.4 mV), but did not modify significantly (*p* > 0.05) the surface charge density of HHPI2. Although an increase in surface charge is generally associated with improved solubility, due to the maximization of the protein–protein repulsive forces and the ion-dipole interactions with the water molecules [29], our findings did not show such a trend. It is therefore deduced that HPI solubility was governed by a balance of the electrostatic and hydrophobic forces rather than the overall surface charge density alone.

With regard to HPI surface hydrophobicity, as shown in Figure 1C, HPI2 exhibited the highest H_0_ value (46 a.u.), while HPI7 and HPI10 the lowest (5 a.u.), with no significant difference between them (*p* > 0.05). These results corroborate the findings of Tang et al. [30], who assessed the H_0_ of various plant proteins, including soy, pea, lupin, and canola. The higher H_0_ observed at acidic conditions most probably stems from the exposure of the hydrophobic moieties (e.g., aliphatic and aromatic amino acid residues) to the protein surface due to the partial unfolding of tertiary protein structure. However, it is important to note that ANS, being an anionic probe, may interact with positively charged groups at low pH, resulting in an overestimation of the H_0_ of HPI2 [31,32]. Furthermore, heat treatment significantly (*p* < 0.05) increased the surface hydrophobicity of both HHPI7 and HHPI10, which both had values of 10 a.u., most likely due to the exposure of additional hydrophobic groups on the protein surface. In contrast, no significant change (*p* > 0.05) was observed for HHPI2, which nevertheless maintained the highest H_0_ value. A similar increase in surface hydrophobicity under neutral conditions after heat treatment has also been reported for various plant proteins, including soy, pea, and peanut [33,34].

#### 3.1.2. Protein Secondary Structure

**Figure 2:**
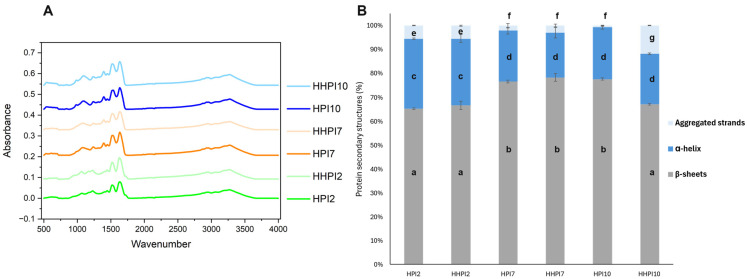
(**A**) HPI samples FTIR. (**B**) Protein secondary structures and their prevalences in percentage (%) as obtained from the peak deconvolution of the amide I region of the samples FTIR. ^a–g^ Different letters denote significant differences according to Tukey’s post hoc means comparison test (*p* < 0.05). Heat-treated samples are here referred to as HHPI.

To assess the effect of pH and heat treatment on the secondary structures of HPI, FTIR of the samples were acquired, and the amide I region peak (1700–1600 cm^−1^) was considered and deconvoluted. It is well-documented that changes in the FTIR spectrum of the amide I region are associated with modifications in the secondary structure conformation of proteins [35]. Amide I peak deconvolution allowed for the identification of three major secondary structure conformations, here presented in Figure 2, and assigned to β-sheet (1630–1623 cm^−1^), α-helix (1660–1650 cm^−1^), and intermolecular or aggregated β-sheet strands (1691–1680 cm^−1^) [11,36]. According to the results presented in Figure 2, HPI samples exhibited predominant β-sheet conformation in all the pHs considered, with a significantly higher (*p* < 0.05) proportion for HPI7 and HPI10 (~77%) compared to HPI2 (~66%). On the other hand, HPI2 had a significantly higher (*p* < 0.05) proportion of α-helix conformations (29% against 21%) and aggregated strands (5.5% against 1.5%) compared to all the other samples. It has been previously shown that the protein extraction conditions are inextricably associated with the secondary structure of hemp protein isolates [11,37]. In their study, Fang et al. [37] reported that alkali extraction/isoelectric precipitation HPI derivatives exhibited a β-sheet-dominant protein secondary structure (~55%) followed by random coil (~16%) and α-helix (~12%) conformations. The prevalence of β-sheet structures was ascribed to the higher content of the HPI in edestin than albumin (79 vs. 21%, respectively) and can explain the moderate water solubility, albeit comparable to that of pea and pulses protein isolates [37]. Moreover, the high resistance of the protein secondary structure to thermal processing was observed under acidic and neutral conditions, as no significant differences (*p* > 0.05) were detected before and after the treatment. On the contrary, in alkaline conditions, heating resulted in a significant increase (*p* < 0.05) in the amount of aggregated β-sheet strand structures at the expense of β-sheets, indicating the unfolding of the native ordered structures and their re-assembly into highly stable aggregates.

#### 3.1.3. HPI Dispersions Particle Size

**Figure 3:**
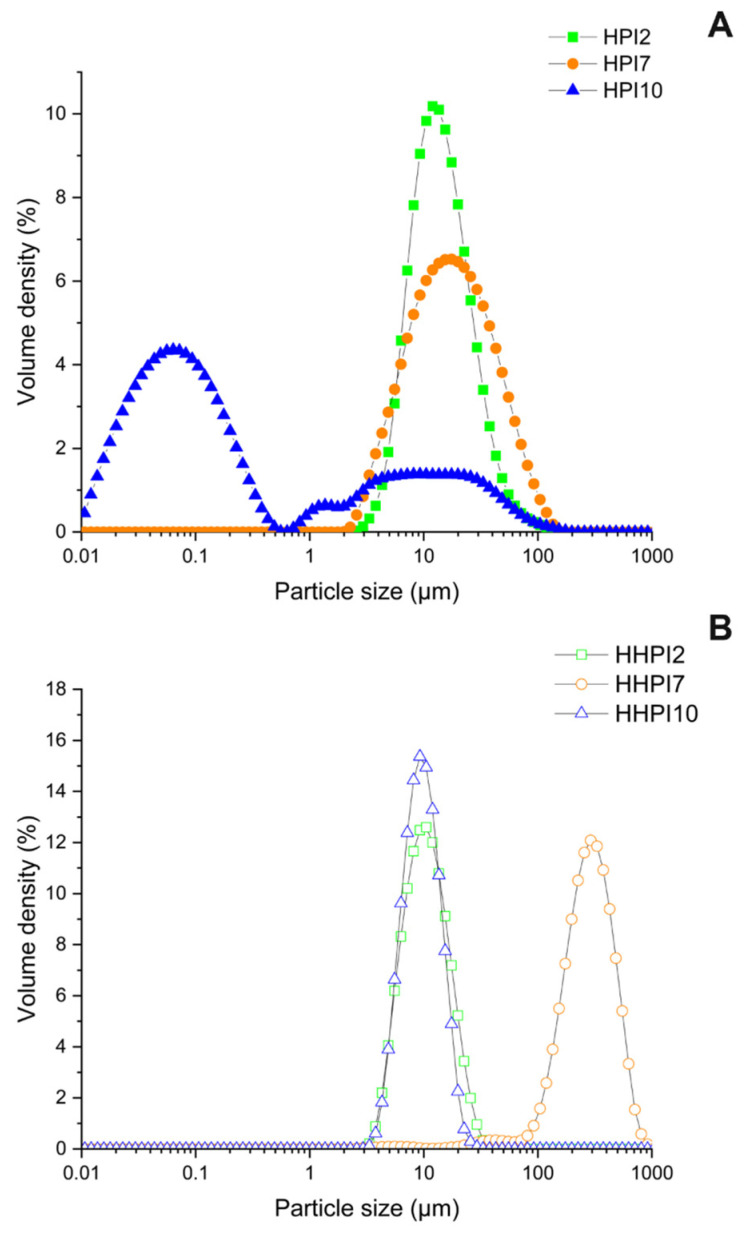
Particle size distribution of HPI dispersion samples before (**A**) and after (**B**) heat treatment.

Figure 3 shows the particle size distributions of the aqueous HPI dispersions before and after heat treatment. As seen in Figure 3A, HPI2 and HPI7 exhibited similar monomodal distributions, with particle size (d_4,3_) peaking at 13.5 µm and 17.5 µm, respectively, while a bimodal distribution, as evidenced by the nano- and above-micron particle populations at 0.1 µm and a lower at 10 µm, was found for HPI10 dispersions. In particular, the finer soluble protein aggregates obtained at alkaline conditions are ascribed to the higher net surface charge and the lower hydrophobicity of the HPI soluble oligomers preventing their aggregation via short range forces, i.e., hydrogen bonding or van der Waals forces [38]. Heat treatment of the HPI dispersions (Figure 3B) shifted the particle size distribution peaks of HHPI10 to around 10 µm, reaching a similar particle size distribution to HPI2, and markedly increased the size of HHPI7, causing a major shift from 17.5 to over 200 µm, thus resulting in the formation of large aggregates. The distinct aggregation behavior observed at pH 7 and pH 10 highlights the combined influence of pH-dependent protein charge and thermal treatment on aggregation mechanisms. At pH 7, hemp proteins carry a moderate net charge, resulting in limited electrostatic repulsion between molecules. Upon heating, partial unfolding exposes hydrophobic regions that promote intermolecular association, primarily through hydrophobic interactions and native-like clustering. This leads to a pronounced increase in particle size without significant changes in secondary structure (accordingly to FTIR results), indicating aggregation dominated by supramolecular associations rather than extensive secondary-structure rearrangement. In contrast, at pH 10, hemp proteins are highly negatively charged, which increases electrostatic repulsion and promotes greater molecular unfolding and conformational flexibility. Upon heating, these conditions favor the formation of intermolecular β-sheet structures once electrostatic barriers are locally overcome, resulting in the simultaneous increase in aggregated β-sheet strands and particle size, indicating the formation of larger, more ordered, and structurally stabilized protein aggregates [39,40]. Nevertheless, HHPI2 did not show any shift in its particle size distribution, confirming its resistance to thermal processing.

### 3.2. Interfacial Adsorption and Rearrangement Properties

**Figure 4:**
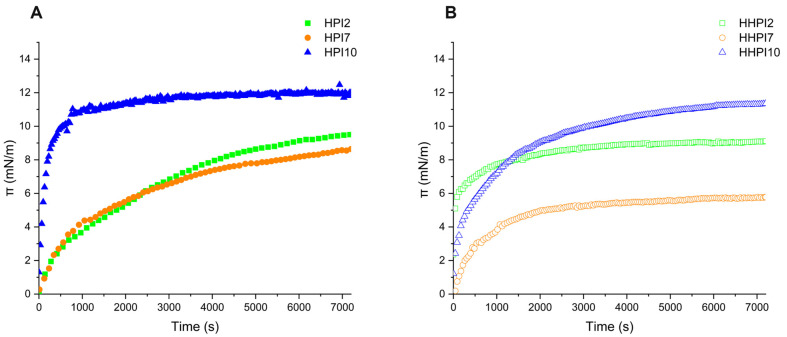
Interfacial pressure π (mN/m) of HPI samples as a function of time (s) before (**A**) and after heating treatment (**B**).

The evolution of interfacial pressure values (π) as a function of pH over a period of 7200 s is presented in Figure 4A. Overall, the presence of proteins was associated with an increase in the interfacial pressure due to their ability to migrate and adsorb onto the interface between the two immiscible phases. In addition, the rate of increase in π values declined over time, a typical behavior previously reported by Graham et al. [20], which they attributed to surface saturation by the protein increasing the energy barrier for further adsorption.

In particular, the interfacial pressure of HPI7 and HPI2 steadily increased and eventually reached stable values around 8 to 9 mN/m, respectively, with no significant difference between them (*p* > 0.05). On the other hand, HPI10 showed a rapid increase in π values during the first 500 s, ultimately reaching the significantly higher value (*p* < 0.05) of 12 mN/m at the end of measurement. This effect stems from the presence of the sub-micron-sized HPI10 soluble aggregate population that can be rapidly adsorbed to the oil-water interface, leading to an effective reduction in its interfacial tension and, thus, an increase in its osmotic pressure. This interfacial behavior is consistent with the findings of Chang et al. [32], who correlated the ability of different plant proteins to adsorb and reach higher interfacial pressure with their physicochemical properties, suggesting that optimal effectiveness occurs with high net charge and moderate hydrophobicity. In this context, HPI10 dispersions (high surface charge, low hydrophobicity) were more effective in increasing interfacial pressure than their HPI2 counterparts (high surface charge, high hydrophobicity). Interestingly, HPI7 achieved a similar moderate π increase to HPI2, a result likely governed by their comparable particle size, overriding their different charge and hydrophobicity profiles.

Furthermore, as can be seen in Figure 4B, heat treatment did not modify (*p* > 0.05) the ability of both HHPI2 and HHPI10 to increase interfacial pressure, while it negatively affected HHPI7 (*p* < 0.05), reaching a lower pseudo-equilibrium π value of 5.7 mN/m. This behavior is attributed to the heat-induced changes in HPI’s physicochemical properties. Although heating increased the charge and hydrophobicity of both HHPI7 and HHPI10, HHPI7’s large particle size and low soluble protein fraction significantly impaired its interfacial adsorption and pressure increase. Conversely, HHPI10 maintained effective adsorption despite a size increase, reaching final particle dimensions similar to HPI2. However, the differences in the evolution pattern of interfacial pressure during the initial migration stage imply that heating is highly influential on the intermolecular interplay between protein molecules at the interface, which ultimately impacts their kinetics of adsorption [41].

**Figure 5:**
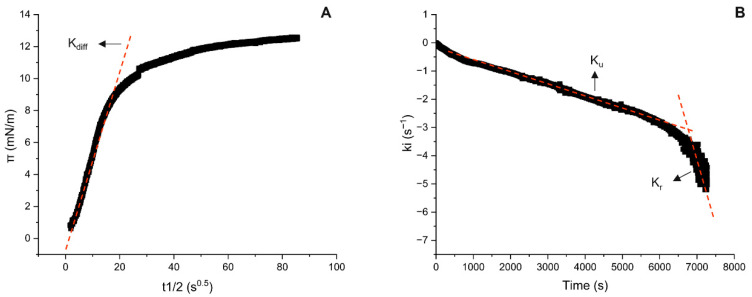
Linear region of the plot of pressure π (mN/m) versus t^1/2^ (s^0.5^) to obtain the constant of diffusion k_diff_ (**A**) and the two linear regions of the plot of ln[(π_7200_ − π_t_)/(π_7200_ − π_0_)] to obtain the constants of unfolding and rearrangement k_u_ and k_r_ (**B**).

The initial stage of protein adsorption involves diffusion from the bulk to the interface. The diffusion-controlled rate is given by the diffusion constant, k_diff_, corresponding to the slope of the linear region in the early plot of interfacial pressure (π) versus the square root of time ($\sqrt{t})$ (Figure 5A). As given in Table 1, the k_diff_ values were comparable for HPI2 and HPI7 samples, whereas HPI10 exhibited a significantly higher value (*p* < 0.05). While previous studies have demonstrated that protein concentration and physicochemical properties primarily govern diffusion and adsorption, our results suggest that, in this case, protein particle size was the dominant driving force [13,21]. According to the Stokes–Einstein diffusion model, the diffusion coefficient is inversely proportional to the cubic root of the particle’s hydrodynamic volume [6,42]. In this context, the presence of the submicron sized soluble aggregates in HPI10 dispersions promoted faster diffusion from the bulk to the interface than in the neutral and acidic counterparts. This is also supported by the diffusion-controlled time t_diff_, which was reduced from ~650 s to ~250 s in alkaline conditions. Regarding heat treatment, it showed a significant difference (*p* < 0.05) for HHPI10, which increased the particle size contributed to reduce its adsorption kinetics, showing no more difference with protein molecules in neutral and acidic conditions, even though its diffusion period was increased to ~800 s. Also HHPI2 presented a significantly higher k_diff_ value and reduced diffusion period from ~600 s to ~200 s, which we hypothesized was due to an improvement of intermolecular interactions, such as the formation of additional hydrogen bonding as a result of protonation of carboxyl groups in proteins and the loss of localized electrostatic interactions of the ionized carboxylates, as well as the formation of disulfide bonds as described by Kella and Kinsella and Wang et al. [43,44], which could also explain HHPI2’s resistance to heat, as described in this study.

### 3.3. Protein Unfolding and Rearrangement at the o/w Interface

After protein migration, the rate of adsorption gradually decreases due to protein saturation at the interface. The second and third steps of adsorption are represented by protein unfolding and conformational rearrangement, the rate of which can be evaluated from the constants k_u_ and k_r_, respectively [6]. The k_u_ and k_r_ values, calculated using the method presented in Figure 5B, are summarized in Table 2. All the samples showed no significant differences (*p* > 0.05) for the unfolding constant k_u_, indicating that, regardless of pH and heat treatment, the proteins exerted a similar unfolding behavior, possibly because of similar protein–protein interaction development once the molecules adsorbed and reached the equilibrium at the interface.

On the other hand, rearrangement constant values k_r_ showed some differences, presenting a higher value (*p* < 0.05) in neutral conditions compared to both acidic and alkaline, probably indicating weaker protein–protein interactions, which favored an easier rearrangement. Moreover, heat treatment decreased the magnitude of k_r_ values for both HHPI2 and HHPI7, in agreement with the findings on soy glycinin nanoparticles at the o/w interface described by Liu and Tang [45]. The authors associated this behavior with a lower ease of structural rearrangement of the adsorbed protein molecules after thermal processing, which could be due to increased thermodynamic stability with lower free energy, as also described by Jahn and Radford [46]. However, in this study, HHPI10’s value increased after heating, reaching similar values to HPI2 and HHPI7, thus indicating a faster rearrangement at the interface and possibly different intermolecular interactions between the adsorbed molecules. To further explore if protein–protein interactions were been influenced by the different environmental and processing conditions, we proceeded with the analysis of the rheological properties of the adsorbed interfacial protein layer.

### 3.4. Interfacial Rheology

**Figure 6:**
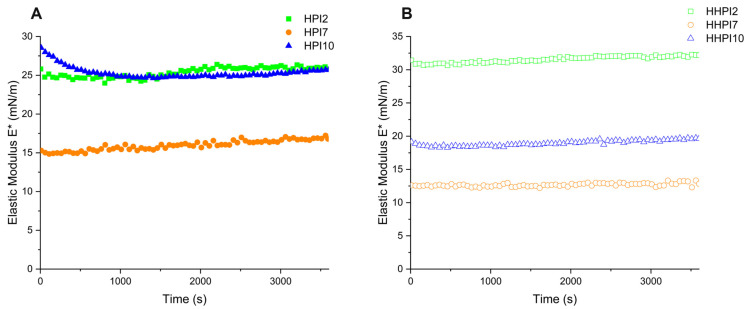
Complex dilatational viscoelastic modulus E^*^ (mN/m) of HPI samples as function of time (s) before (**A**) and after heating treatment (**B**).

It is well-established that protein adsorption and rearrangement at the interface leads to the formation of an intermolecular network whose rheological characteristics are governed by the physicochemical properties of protein molecules, which affect their intermolecular interactions [47]. To better understand the rheological properties of HPI at the o/w interface, oscillatory dilatational deformation tests were performed once the proteins attained the equilibrium state, and the evolution of the dilatational viscoelasticity modulus E^*^ as a function of time was monitored (Figure 6A). All samples were able to form a viscoelastic film at the interface, increasing E^*^ value of the pure system (water with no added proteins) from 0 mN/m to 15–26 mN/m, indicating that protein molecules are able to form a solid-like cohesive network after adsorbing and rearranging at the interface [48]. Moreover, the E^*^ value did not present a large amplitude dependency over the tested range, suggesting that protein–protein interactions remained intact under the applied surface area changes. This behavior is fundamental for the ability of a biopolymer to stabilize an emulsion colloidal system over time, avoiding phase separation and, consequently, collapse. In this study, HPI7 showed the lowest E^*^ value of 15 mN/m over the whole considered period of time of 3600 s, indicating a less structured network at the interface, while both HPI2 and HPI10 stabilized at E^*^ values of 25 mN/m, forming a stronger viscoelastic film, which could result in the better long-term stability of the emulsion. These differences in the strength of the viscoelastic membrane are thought to be an expression of the physicochemical properties of the proteins, and, in particular, of their intermolecular interactions (i.e., hydrophobic, van der Waals, etc.). The enhanced viscoelasticity of the HPI dispersions at pH 2 and 10 may be associated with their higher solubility, allowing for a steady adsorption at the interface while surface charge and hydrophobicity at different pHs modify the protein–protein interactions, ultimately affecting the interfacial layer strength and thickness. These findings are in accordance with the interfacial viscoelastic behavior of other plant proteins such as soy, pea, lupin, and canola [32]. Specifically, it has been showcased that these proteins exhibit higher viscoelastic stiffness at acidic conditions (pH 3) compared to neutral conditions (pH 7). In the latter case, no film network was formed, resulting in the fluid-like rheological behavior of the proteins at the interface, most likely due to weakened protein–protein interactions.

The effect of heat treatment on the dilatational viscoelasticity modulus E^*^ of HPI dispersions as function of pH is shown in Figure 6B. Overall, temperature led to changes into interfacial elasticity, indicating alterations in protein–protein interactions and/or protein structures. Heating improved the interfacial elasticity of HHPI2, with its value increasing from 25 to 32.1 mN/m, possibly due to controlled unfolding and enhanced molecular interactions, which corroborate the findings obtained for its adsorption properties. A similar behavior was previously reported for soy proteins heat-treated at 90 °C and was ascribed to the increase in surface hydrophobicity and the formation of disulfide bonds between neighboring proteins [44]. Nevertheless, heat treatment weakened the interfacial network of HHPI7 and HHPI10, as reflected by the reduction in the dilatational viscoelastic modulus E^*^ to 19.6 mN/m and 12.8 mN/m, respectively. These results are ascribed to the increase in surface charge density and mean size of the HHPI7- and HHPI10-soluble aggregates, leading to the weakening of the interfacial layer due to steric hindrances and electrostatic repulsion. On the other hand, no change in either surface charge or soluble aggregates size was observed for HHPI2, whose improved viscoelastic network is presumably associated with the presence of more flexible molecular structures, which permitted the formation of intermolecular junctions through disulfide bonds at the surface of protein molecules. A similar effect was observed by Zhou et al. [6], who associated the increase in surface charge after heating treatment with the formation of a less structured and more elastic protein layer at the interface. A slight decrease in elastic modulus after heating was also described in the case of peanut protein isolate at pH 7, which was attributed to the modification of the structural conformation of protein molecules promoting different intermolecular contacts and conformations of the protein molecules [34].

### 3.5. Emulsifying Properties and Stability

**Figure 7:**
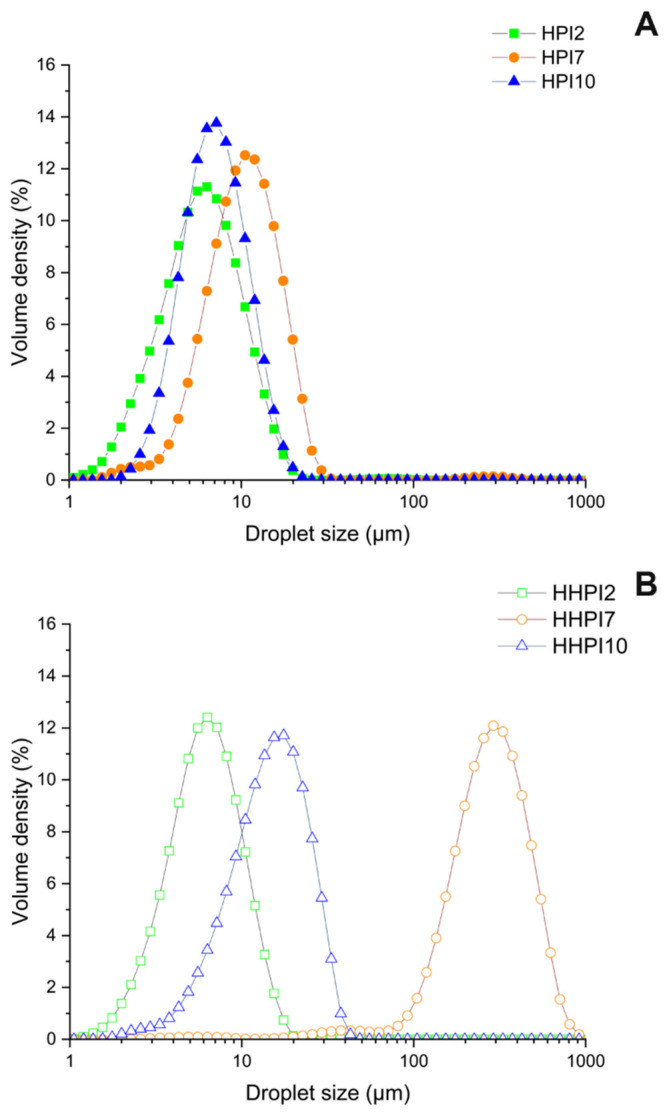
Droplet size distribution of HPI particles oil-in-water (o/w) stabilized emulsions before (**A**) and after (**B**) heat treatment.

The droplet size distributions of the emulsions stabilized by the native and heat-treated HPI at different pH values are illustrated in Figure 7. In all cases, monomodal lipid droplet size distributions with varying spans were observed. HPI2-based emulsions displayed the smallest droplet size (d_4,3_ = 6.7 µm), followed by HPI10 and HPI7 (d_4,3_ = 7.6 and 13 µm, respectively). It has been previously demonstrated that the emulsifying capacity of HPI is inextricably associated with the structural, conformational, and technofunctional aspects of the proteins that are influenced by the extraction and isolation process. In this context, Dapčević-Hadnađev et al. [49] reported that the emulsifying capacity of micellar HPI at pH 3 is significantly better than that of alkali-extracted/pI-precipitated analogs, which was attributed to the higher water solubility and lower degree of denaturation of the former. In addition, the shift from acidic to alkaline conditions has been associated with a significant increase in mean lipid droplet size in the case of legume proteins (soy, pea, lupin) and has been primarily ascribed to the pH-induced changes in their water solubility [32]. In a recent study, Zhou et al. [6] demonstrated that the increased net surface charge density of WPI at highly acidic or alkaline conditions results in the formation of strongly repelling protein layers preventing emulsion destabilization due to coalescence or bridging flocculation. It should be noted that the higher span in the HPI2 o/w emulsion compared to the HPI10 exemplars implies less finely dispersed lipid droplets, most probably due to the excessive protein unfolding and/or higher degree of denaturation. This finds support on the interfacial rheology measurements, where HPI at pH 2 and pH 10 formed interfacial layers with comparable elasticity whereas HPI7 exhibited the lowest elasticity, indicating a greater susceptibility to phase separation over time. Upon heating, a shift toward larger droplet sizes was observed for HHPI10 (d_4,3_ = 17.6 µm) and an even more pronounced increase for HHPI7 (d_4,3_ = 290 µm). The latter implies that the occurrence of coalescence due to the elimination of the electrostatic barrier, leading to bridging flocculation and eventually to the rupture of the interfacial film as the Laplace pressure increases [50]. Nevertheless, thermal processing of HHPI2 dispersions did not modify the lipid droplet size of the o/w emulsions due to their higher interfacial elasticity.

**Figure 8:**
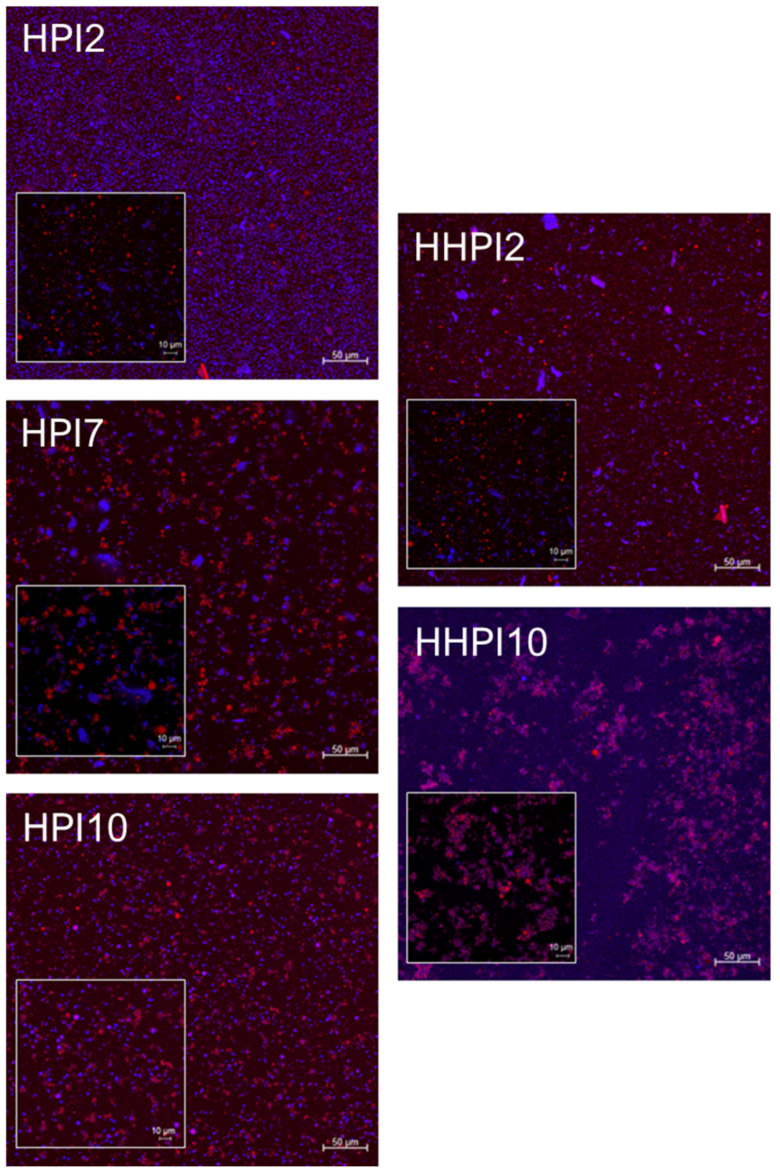
CLSM images of the HPI stabilized o/w emulsions at different pH values before and after heat treatment. Images were acquired at ×20 and ×63 (inlay). Red represents the stained oil phase, while blue represents the stained protein particles.

CLSM assessment of the o/w emulsions (Figure 8) confirmed that the HPI2 stabilized emulsions exerted the finest lipid droplet dispersions compared to the HPI7 and HPI10 counterparts. As illustrated in the 63× CLSM micrographs of the HPI2 o/w emulsions, a densely packed emulsion consisting of highly uniform lipid droplets covered by a thick protein interfacial shell were obtained. In contrast, the HPI7 CLSM images evidenced the occurrence of lipid droplet flocculation and partial coalescence stemming from the weak protein adsorption at the lipid–water interface as result of the protein–protein interactions favored by the suppression of the electrostatic force barrier. Although the HPI10 emulsions exhibited a highly fine-dispersed lipid phase, as in the case of the HPI2 systems, the occurrence of limited flocculation was observed to be in good agreement with the SLS measurements (i.e., right shift in the monomodal lipid droplet size peak). Thermal processing of the HPI dispersions promoted the formation of heat-induced protein aggregates in all cases (CLSM images of HHPI7 were not included in Figure 8 due to excessive protein aggregation and sedimentation). In both HHPI2 and HHPI10 systems, heating resulted in partial coalescence, as implied by the increase in lipid droplet and non-adsorbed protein polydispersity stemming from the partially steric hindered interfacial adsorption of protein material. In keeping with the SLS measurements, the HHPI10 CLSM images evidenced the occurrence of aggregative phase separation, with lipid droplets being immobilized in the aggregated protein-rich microdomains.

The colloidal stability of the HPI stabilized o/w emulsions under accelerated storage isothermal (25 °C) conditions was tested using a LUMiSizer (Supplementary Figure S1). As illustrated in the light transmission spectra (Supplementary Figure S1) HPI2 stabilized emulsions did not show any remarkable difference in light transmission over the entire centrifugation time representing the most stable sample against creaming. According to the calculated colloidal instability indices (CIs) (Table 3), the resistance of the o/w emulsions against creaming was reduced according to the order: HPI2 > HPI10 > HPI7, which is in keeping with the interfacial properties of the HPI dispersions. Thermal processing of the HPI dispersion did not significantly modify the CI of the HPI2-stabilized emulsions (i.e., 0.013 vs. 0.011 for HPI2 and HHPI2, respectively). On the contrary, HHPI7- and HHPI10-based emulsions underwent a steep decrease in the CI values (from 0.707 to 0.239 and 0.685 to 0.257, respectively). However, it should be noted that, in HHPI7, the increase in the light transmission in the partially lipid phase depleted (opaque bottom) phase evidenced sedimentation phenomena due to the presence of large protein aggregates (see also Section 3.1.3).

To gain an insight into the kinetics of the creaming behavior of the emulsions, the time–colloidal instability index data (the HHPI7 based emulsions were excluded due to the sedimentation occurrence) were fitted with the Hill’s model as follows:

(5) $$\mathrm{CI}\;=\;CI_{\max}\frac{t^{\alpha }}{t^{\alpha }\;+{\;\theta }^{\alpha }}$$

where CI and CI_max_ denote the colloidal instability index at t = 600 and t = ∞ (in s), θ represents the time required to reach 50% instability, and α is the cooperativity index (dimensionless) associated with the creaming destabilization phenomena. As shown in Table 3, in the case of HPI7-, HPI10-, and HHPI10-based emulsions, the CI values obtained at the end of the experiment (i.e., t = 600 s) closely approached the CI_max_ values (i.e., CI/CI_max_~0.94 to 0.98) estimated according to the Hill’s model, indicating the attainment of a pseudo-equilibrium state. On the contrary, the HPI2- and HHPI2-based emulsions remained far from equilibrium, with CI/CI_max_ being ~0.11 and 0.34, respectively. In a kinetic context, the HPI2- and HHPI2-based emulsions exhibited the lowest creaming rates (θ > 2900 and 900 s, respectively), confirming the formation of strong elastic interfacial protein films compared to their neutral or alkaline counterparts. In all the emulsion systems, the cooperativity coefficient was higher than unity (α > 1), which is indicative of the acceleration of the creaming process once it commences. Thus, it is assumed that the creaming process is progressively accelerating due to early-stage partial coalescence or bridging flocculation phenomena, which increase lipid droplet size until steric hindrance limits further aggregation. Noteworthily, the HPI2-based emulsion had the lowest α values, indicating a better solvation of the proteins at the interfaces and the formation of better protective interfacial steric barriers compared to the HPI7 and HPI10 exemplars. Thermal processing resulted in a significant increase in the α values, suggesting the deterioration of the steric barrier capacity of the interfacial films formed, in agreement with our interfacial measurements. In this context, the association of extrinsic parameters such pH and heating play a modulatory role on emulsion stability kinetics via their ability to tune the particle size, surface charge, water solvation affinity, and hydrophobicity of the adsorbed protein molecules.

## 4. Conclusions

This study investigated the effect of extrinsic parameters (pH and heat treatment) on the physicochemical, interfacial, and emulsifying properties of hemp seed protein isolate (HPI) dispersion in order to increase its employability as alternative protein source in food formulations. Overall, the findings demonstrate that pH plays a crucial role in determining the physicochemical properties of HPI dispersions and, consequently, their interfacial and emulsifying properties. In general, both at pH 2 and 10, HPI presented optimal physicochemical properties and overall high soluble fraction, which resulted in better interfacial properties, allowing for improved adsorption and intermolecular interactions at the oil–water interface. In particular, pH 10 promoted a faster interfacial adsorption of the protein molecules due to submicron particle size, while pH 2 allowed for the development of a strong interfacial layer due to high hydrophobicity and surface charge, overall providing optimal intermolecular interactions. In contrast, pH 7 presented a weaker interfacial elasticity, most likely due to poor solubility, which ultimately led to reduced emulsion stability as presented by the large oil droplet size distribution and high creaming index. Heat treatment enhanced emulsion stability at pH 10 due to structural modifications and increased particle size distribution, surface charge, and hydrophobicity, but did not affect dispersions at pH 2, which showed great temperature resistance and the finest lipid droplet dispersion, and thus the highest emulsifying stability, while further decreasing the functionality of dispersions at pH 7 due to increased particle size, resulting in precipitation, significantly increasing oil droplet size and the coalescence destabilization effect. These findings offer valuable insights on the interfacial behavior of oil in water in HPI dispersions, highlights their potential as natural, clean-label food emulsifiers under diverse extrinsic physicochemical conditions, and ultimately should be considered in the context of increased application and valorization of plant-based functional ingredients.

## Figures and Tables

**Table 1:** Characteristic dynamic parameters for diffusion of HPI dispersions to the o/w interface as a function of pH before and after heat treatment.

Sample	K_diff_ (mN m^−1 ^s^−0.5^)	R^2^	t_diff_ (s)
HPI2	0.14 ± 0.01 ^a^	0.99	648 ± 20 ^a^
HHPI2	0.22 ± 0.01 ^b^	0.99	220 ± 07 ^b^
HPI7	0.14 ± 0.04 ^a^	0.98	618 ± 12 ^a^
HHPI7	0.16 ± 0.00 ^a^	0.99	625 ± 11 ^a^
HPI10	0.57 ± 0.06 ^c^	0.99	248 ± 25 ^b^
HHPI10	0.16 ± 0.05 ^a^	0.99	809 ± 14 ^c^

k_diff_ represents the slope of the linear region in the π–t^1/2^ plot, taken as the rate of initial diffusion-controlled migration. t_diff_ represents the period during which diffusion controls the kinetics of adsorption of HPI at the o/w interface. ^a–c^ Different letters denote significant differences according to Tukey’s post hoc means comparison test (*p* < 0.05). Heat-treated samples are here referred to as HHPI.

**Table 2:** Characteristic dynamic parameters for the unfolding (k_u_) and rearrangement (k_r_) of the HPI dispersions at the o/w interface as a function of pH before and after heat treatment.

Sample	K_u_ × 10^4^ (s^−1^)	R^2^	K_r_ × 10^4^ (s^−1^)	R^2^
HPI2	5.15 ± 0.65 ^a^	0.99	9.95 ± 1.45 ^a^	0.96
HHPI2	4.7 ± 0.4 ^a^	0.98	4.05 ± 1.55 ^b^	0.96
HPI7	3.95 ± 0.35 ^a^	0.98	14.85 ± 3.65 ^c^	0.96
HHPI7	5.7 ± 0.0 ^a^	0.96	6.5 ± 0.0 ^ab^	0.96
HPI10	4.7 ± 0.2 ^a^	0.98	6.15 ± 0.05 ^ab^	0.96
HHPI10	4.25 ± 0.95 ^a^	0.98	9.9 ± 0.0 ^a^	0.96

^a–c^ Different letters denote significant differences according to Tukey’s post hoc means comparison test (*p* < 0.05). Heat-treated samples are here referred to as HHPI.

**Table 3:** Colloidal instability index (CI) and kinetic colloidal instability parameters, as calculated according to Hill’s model (Equation (5)), for HPI samples as a function of pH before and after heat treatment. Heat-treated samples are here referred to as HHPI. nd, not detected.

Sample	CI_t=600s_	CI_max,t=∞_	θ	α	R^2^
HPI2	0.013	0.115	2940	1.29	0.99
HHPI2	0.011	0.032	901.4	1.45	0.99
HPI7	0.707	0.746	109.8	1.82	0.99
HHPI7	0.239	nd	nd	nd	nd
HPI10	0.685	0.696	89.20	1.95	0.99
HHPI10	0.257	0.265	304.5	2.31	0.99

## Data Availability

The original contributions presented in this study are included in the article and Supplementary Materials. Further inquiries can be directed to the corresponding authors.

## Supplementary Materials

The following supporting information can be downloaded at https://www.mdpi.com/article/10.3390/foods15020257/s1, Figure S1: (A) Light transmission versus position of the sample in the sample vial of HPI samples over a period of time of 600 s, here depicted in different colors from red to green; (B) Instability index evolution over time for HPI samples.
