# Supplementary material for: Influence of pH and Heat Treatment on the Physicochemical, Interfacial, and Emulsifying Properties of Hemp Seed Protein Dispersions
Source: Foods. 2026 Jan 10;15(2):257. doi: 10.3390/foods15020257 (PMC12839807; doi:10.3390/foods15020257)
Supplement: Supplementary file 1 [file foods-15-00257-s001.zip › foods-4055191-supplementary.pdf]

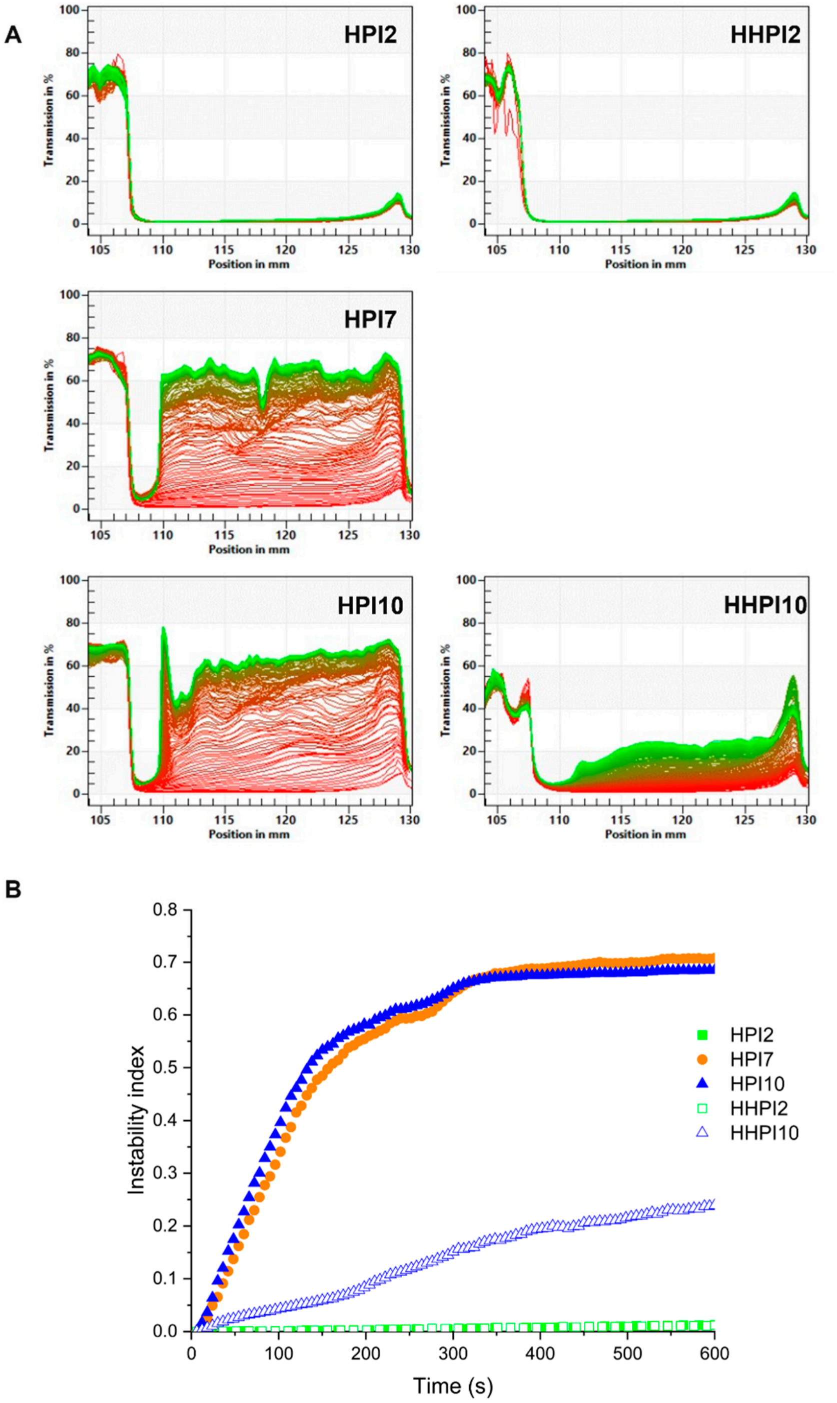

Supplementary Figure S1. (A) Light transmission versus position of the sample in the sample vial of HPI samples over a period of time of 600 s, here depicted in different colors from red to green; (B) Instability index evolution over time for HPI samples.
